# Association between Tumor Size and Bilateral Involvement in Papillary Thyroid Carcinoma

**DOI:** 10.1155/2016/8470252

**Published:** 2016-04-24

**Authors:** Suna Erkilic, Fatih Celenk, Zehra Bozdag

**Affiliations:** ^1^Department of Pathology, Faculty of Medicine, Gaziantep University, 27310 Gaziantep, Turkey; ^2^Department of Otorhinolaryngology, Faculty of Medicine, Gaziantep University, 27310 Gaziantep, Turkey

## Abstract

*Background*. Tumor multifocality and bilaterality of papillary thyroid carcinoma (PTC) are important factors when selecting the most appropriate surgical procedure. The aim of this study was to assess the bilaterality rate in PTC and the relationship between the tumor size and bilaterality.* Materials and Methods*. Thyroidectomy specimens with a diagnosis of PTC were retrospectively reviewed in the Pathology Department of a tertiary care medical center. Specimens were divided into three groups according to the size of the primary and contralateral tumor foci. Tumors less than or equal to 1 cm in each lobe were included in group 1. Group 2 consisted of tumors greater than 1 cm in one lobe and less than 1 cm in the other lobe. Tumors greater than 1 cm in each lobe were included in group 3.* Results*. We identified 868 total thyroidectomy specimens with a diagnosis of PTC between 2001 and 2011. Of these cases, both thyroid lobes were involved in 262 cases (32%). There were 109 (42%), 121 (46%), and 32 cases (12%) in group 1, group 2, and group 3, respectively.* Conclusion*. Bilaterality is frequent in PTC and is not related to tumor size. Accordingly, the high frequency of bilateral disease in PTC should be kept in mind when determining the extent of the surgical procedure.

## 1. Introduction

Papillary thyroid carcinoma (PTC) is the most common thyroid malignancy. Most of the PTCs have an indolent course and a favourable prognosis [1]. The frequency of multifocal involvement of the thyroid gland in patients with PTC varies widely between 18% and 87% [2]. Multifocal disease is also common in papillary thyroid microcarcinoma being identified in 38% of the cases [3]. Multifocal involvement of the thyroid gland in patients with PTC is associated with poorer prognosis and increased risk of lymph node metastasis and distant metastasis [4, 5]. There are several factors that increase the risk of contralateral disease in patients with PTC including large tumors, being above 45 years of age, and involvement of lymph node(s) [1]. Thus, considering the tumor multifocality and especially bilaterality of papillary thyroid carcinoma (PTC) is important while selecting the most appropriate surgical procedure. The aim of this study was to assess the bilaterality rate in PTC and the relationship between the tumor size and bilaterality.

## 2. Materials and Methods

Thyroidectomy specimens with a diagnosis of PTC were retrospectively reviewed in the Pathology Department of a tertiary care medical center. Data including extent of the surgery, tumor size, and bilaterality were collected. Bilateral involvement was defined as at least 1 tumor focus in the contralateral lobe of the primary tumor.

Total and completion thyroidectomy specimens were included in the study; unilateral lobectomy, subtotal thyroidectomy, and near-total thyroidectomy specimens were excluded. The specimens were evaluated for bilaterality and tumor size. Specimens were divided into three groups according to the size of the primary and contralateral tumor foci. Tumors less than or equal to 1 cm (microcarcinoma) in each lobe were included in group 1. Group 2 consisted of tumors greater than 1 cm in one lobe and less than 1 cm in the other lobe. Tumors greater than 1 cm in each lobe were included in group 3.

## 3. Results

We identified 868 total thyroidectomy specimens with a diagnosis of PTC between 2001 and 2011. Of these cases, both thyroid lobes were involved in 262 cases (32%). There were 109 (42%), 121 (46%), and 32 cases (12%) in group 1, group 2, and group 3, respectively. In group 1, 46 cases had a tumor size less than or equal to 0.5 cm.  Table 1 summarizes the distribution of the bilateral cases according to primary tumor size.

## 4. Discussion

Multifocality and bilaterality in PTC cases are not uncommon. Gerfo et al. [6] found bilateral disease in 32% of the cases and reported the incidence of multicentric disease as 50%. Pacini et al. [7] evaluated tumor bilaterality in patients initially treated with partial thyroidectomy for PTC and found one or more foci of PTC at histology of completion thyroidectomy in 44% of the patients. The rate of bilateral tumor was not different when patients were analyzed according to the classification of low or high risk. Grigsby et al. [1] investigated the rate of contralateral PTC and found that 41% of the patients had PTC in the contralateral lobe. There was no difference in the rate of contralateral disease in low-risk patients and there were no significant differences between patients with or without contralateral disease with respect to primary tumor size. Pitt et al. [8] performed either completion or total thyroidectomy in 228 patients with PTC and evaluated contralateral tumor rates. They observed no differences in the rate of contralateral disease in patients with primary PTC with tumor size ≥1 cm compared with those having disease with tumor size <1 cm. 27% of the patients with tumors <0.5 cm also had contralateral disease. They found multifocality as the only factor predictive of contralateral PTC in patients with microcarcinoma.

Connor et al. [9] investigated the risk factors associated with papillary thyroid microcarcinoma (PTMC) involving the thyroid lobes bilaterally at the time of diagnosis and 20% of the patients had bilateral involvement. Although the bilaterality was frequent in case of multifocality, tumor size was not a predictive factor. Zhou et al. [10] performed total thyroidectomy in 211 PTMC patients and reported multifocality and tumor size ≥7 mm as independent predictive factors for bilaterality.

According to the American Thyroid Association Guideline, thyroid lobectomy is recommended for patients with thyroid cancer with tumor size <1 cm [11]. The guideline also recommends bilateral or a unilateral procedure for patients with thyroid cancer with tumor sizes >1 cm and <4 cm without extrathyroidal extension and without clinical evidence of any lymph node metastases. However, we demonstrated that approximately one-third of the PTCs involve both thyroid lobes. Among cases with bilateral involvement, 42% had tumor size less than or equal to 1 cm in each lobe. Only 12% of the bilateral cases had a tumor size greater than 1 cm. These findings suggest that bilateral involvement is not associated with tumor size and even PTCs with tumor size less than 0.5 cm may show bilaterality.

## Figures and Tables

**Table 1:** Distribution of bilateral cases according to primary tumor size.

Specimen groups	Definition	Number of specimens	Percentage (%)
Group 1	Tumor foci less than or equal to 1 cm in each lobe (microcarcinoma)	109	42
Group 2	Tumor foci greater than 1 cm in one lobe and less than 1 cm in the other lobe	121	46
Group 3	Tumor foci greater than 1 cm in each lobe	32	12
